# Supplementary material for: Resolving the full spectrum of human genome variation using Linked-Reads
Source: Genome Res. 2019 Apr;29(4):635–45. doi: 10.1101/gr.234443.118 (PMC6442396; doi:10.1101/gr.234443.118)
Supplement: Supplemental Material [file supp_gr.234443.118_Supplemental_longranger-2.2.zip › longranger-2.2.tar.bz2!/lr2.2-public/lib/stan/cmdstan/stan/doc/api/html/array__builder_8hpp_source.html]

Stan: src/stan/math/matrix/array\_builder.hpp Source File


|  |  |
| --- | --- |
|  | Stan  2.5.0  probability, sampling & optimization |


- Main Page
- Related Pages
- Namespaces
- Classes
- Files

- File List
- File Members

All Classes Namespaces Files Functions Variables Typedefs Enumerations Enumerator Friends Macros Pages

- src
- stan
- math
- matrix

array\_builder.hpp

Go to the documentation of this file.

1 #ifndef STAN\_\_MATH\_\_MATRIX\_\_ARRAY\_BUILDER\_HPP

2 #define STAN\_\_MATH\_\_MATRIX\_\_ARRAY\_BUILDER\_HPP

3

4 #include <vector>

5 #include <stan/math/matrix/promoter.hpp>

6

7 namespace stan {

8  namespace math {

9

15  template <typename T>

16  struct array\_builder {

17  std::vector<T> x\_;

18  array\_builder() : x\_() { }

19  template <typename F>

20  array\_builder& add(const F& u) {

21  T t;

22  promoter<F,T>::promote(u,t);

23  x\_.push\_back(t);

24  return \*this;

25  }

26  std::vector<T> array() {

27  return x\_;

28  }

29  };

30

31  }

32 }

33 #endif

stan::math::array\_builder::x\_

std::vector< T > x\_

**Definition:** array\_builder.hpp:17

stan::math::array\_builder::array

std::vector< T > array()

**Definition:** array\_builder.hpp:26

stan::math::array\_builder::add

array\_builder & add(const F &u)

**Definition:** array\_builder.hpp:20

stan::math::array\_builder::array\_builder

array\_builder()

**Definition:** array\_builder.hpp:18

promoter.hpp

stan::math::array\_builder

Structure for building up arrays in an expression (rather than in statements) using an argumentchaini...

**Definition:** array\_builder.hpp:16

stan::math::promoter::promote

static void promote(const F &u, T &t)

**Definition:** promoter.hpp:15


---

[ Stan Home Page ]

*© 2011–2014,
Stan Development Team.*
